# Numerical Simulation of the Impact of the Heat Source Position on Melting of a Nano-Enhanced Phase Change Material

**DOI:** 10.3390/nano11061425

**Published:** 2021-05-28

**Authors:** Tarek Bouzennada, Farid Mechighel, Kaouther Ghachem, Lioua Kolsi

**Affiliations:** 1 Mechanics of Materials & Plant Maintenance Research Laboratory, (LR3MI), Mechanical Engineering Deprtment, Faculty of Engineering, Badji Mokhtar University, P.O. Box 12, Annaba 23052, Algeria; farid.mechighel@univ-annaba.dz; 2 Department of Industrial Engineering and Systems, College of Engineering, Princess Nourah Bint Abdulrahman University, Riyadh 84428, Saudi Arabia; kgmaatki@pnu.edu.sa; 3 College of Engineering, Mechanical Engineering Department, Ha’il University, Ha’il City 81481, Saudi Arabia; 4 Laboratory of Meteorology and Energy Systems, University of Monastir, Monastir 5000, Tunisia

**Keywords:** NEPCM, melting, nanoparticles, heat transfer, phase change, comsol-multiphysics

## Abstract

A 2D-symmetric numerical study of a new design of Nano-Enhanced Phase change material (NEPCM)-filled enclosure is presented in this paper. The enclosure is equipped with an inner tube allowing the circulation of the heat transfer fluid (HTF); n-Octadecane is chosen as phase change material (PCM). Comsol-Multiphysics commercial code was used to solve the governing equations. This study has been performed to examine the heat distribution and melting rate under the influence of the inner-tube position and the concentration of the nanoparticles dispersed in the PCM. The inner tube was located at three different vertical positions and the nanoparticle concentration was varied from 0 to 0.06. The results revealed that both heat transfer/melting rates are improved when the inner tube is located at the bottom region of the enclosure and by increasing the concentration of the nanoparticles. The addition of the nanoparticles enhances the heat transfer due to the considerable increase in conductivity. On the other hand, by placing the tube in the bottom area of the enclosure, the liquid PCM gets a wider space, allowing the intensification of the natural convection.

## 1. Introduction

The recent industrial revolution required a huge amount of energy, where petrol and gas have been the available energy sources for many years. However, this kind of energy source will surely vanish after few years, regardless of the environmental considerations. Humanity is obligated to find new energy sources, such as solar energy [1], that are renewable and environmentally friendly, but there is an issue with this kind of energy, which is the ability to store heat for a long period to be used when needed. In recent years, phase change materials (PCMs) have become the best choice to store thermal energy because of their high thermal capacity; however, the problem with these materials is their poor thermal conductivity.

The weakness of PCM thermal conductivity is the main obstacle during the process of charging/discharging energy in thermal energy storage systems (TES) [2]. Investigators have suggested and applied several techniques such as adding high conductive fins [3,4] and nanoparticles into the PCM [5,6], where nanoparticles can be used to enhance heat distribution/dissipation due to their high thermal conductivities. Nanofluids can be used in heat exchanger systems [7], where the heat is dissipated via the liquid. The addition of nanoparticles is considered to be an innovative and non-destructive solution to enhance the heat transfer rate. However, a high volume fraction of nanoparticles leads to higher viscosities that cause a significant pressure drop, decreased pumping power, and viscous irreversibility. The same thing can happen with PCM-thermal charge/discharge applications when free convective motions (buoyancy force) occur within the molten region.

Natural convection heat transfer during PCM melting and solidification has been the subject of several numerical and experimental studies [8,9,10]. These studies showed that free convection has an important influence on solidification/melting processes (charging/discharging). Meanwhile, the melting process in the presence of free convection provides higher heat transfer rates compared to the solidification process, as concluded by Bouzennada et al. [8].

Kean et al. [11] performed a numerical simulation on the enhancement heat transfer rate of PCM (paraffin wax) mixed with nanoparticles during the melting process. The study was realized for various volume fractions of nanoparticles. It has been concluded that the melting rate can be improved with low nanoparticle volume fractions. Arasu and Mujumdar [12] numerically studied the pattern of the heat transfer during the melting process in a Nano PCM (paraffin wax with Al_2_O_3_) within a square cavity. The study highlights the impact of the concentration of nanoparticles and the heating position. The authors mentioned that the melting time is increased by adding the Al_2_O_3_ nanoparticles for all the considered heating positions; this is due to the increase of the viscous effect that degrades the free convection’s role. Further, the melting time was faster with the vertical-wall-heating case compared to the bottom-wall-heating case; however, the authors did not clarify the reason for this. It may happen due to the fact that the heat transfer by free convection is better when the heat is supplied vertically. A similar configuration was conducted by Ebrahimi and Dadvand [13] by studying the melting performance and the thermal distribution of an NEPCM inside a square enclosure. They considered different arrangements of two heat source-sink pairs attached on the vertical sidewalls. The authors found that the highest liquid fraction was obtained with alternately placed heat sources. In addition, they mentioned that a nanoparticle concentration of 2% produced the maximum melting rate. Therefore, from the two previous works, it can be concluded that the heating position has an important influence on heat transfer. This fact must be taken into consideration as well as the influence of adding nanoparticles.

Bondareva et al. [14] carried out a numerical study to investigate the behavior of a PCM (n-octadecane) inside a finned heat sink while adding nanoparticles. It was found that, by adding nanoparticles, the melting process was faster at the initial stage, then, once the convection become the dominated mode, the melting time was increased by reducing the volume fraction of the nanoparticles compared to the pure PCM. Additionally, the authors emphasized that, once the nanoparticle diameter increases, the conduction increases, and the viscosity increases as well, and this makes understanding the effect of nanoparticle amount/diameter on natural convection complicated. Additionally, Bondareva et al. [15] carried out a numerical investigation using paraffin as a PCM with added nanoparticles that filled a rectangular cavity. The PCM cavity was heated from the bottom wall. This investigation studied the influence of the inclination angle of the enclosure and the nanoparticle concentration on the melting process for various Rayleigh numbers. The results have proven that changing the inclination angle by more than 90° makes the flow weaker, and the nano PCM (PCM mixed with nanoparticles) melts faster than in the case of pure paraffin, but on the other hand, it was found that, at high Rayleigh values, increasing the nanoparticle concentration increases the melting time due to the increase of the dynamic viscosity of the liquid PCM, hence the flow velocity decreases and the benefit of free convection degrades. The same results were found in Arasu and Mujumdar’s work [12], where the high quantity of nanoparticles decreased the melting rate. The authors mentioned that the reason for that comes from the increase in the liquid PCM viscosity. Although the literature indicates that the use of nanoparticles improves the heat transfer performance when the conduction is the dominant mode, these elements may have an undesirable effect, so the concentration of nanoparticles should be taken into account.

Hosseini et al. [16] have made a 2D-axisymmetric numerical study of melting a nano-enhanced phase change material (NEPCM) in a cylindrical shell, where the PCM is between two co-axial tubes. The R50 was used as the PCM and copper as the nanoparticles. The paper investigated the influence of nanoparticle volume fraction on the melting rate. The results showed that the melting rate increases by increasing the dispersion of nanoparticles, where the heat distribution into the PCM has been enhanced, making the melting rate accelerate.

The nanoparticle effect has also been analyzed during the solidification process. Some of the studies related to this subject are described in the following paragraphs.

Sheikholeslami et al. [17] numerically investigated the heat transfer during the solidification of NEPCM using different CuO nanoparticle shapes (Spherical, Platelet, Cylinder, and Brick), which were dispersed in water (used as PCM), while changing the length of the fins. The authors concluded that the solidification time may diminish by using platelet nanoparticles. Furthermore, increasing the length of the fins improved the conduction mode; consequently, the solidification rate was improved. Du et al. [18] carried out a 3D numerical study to investigate the heat transfer within paraffin (PCM) mixed with copper nanoparticles inside a cylindrical tank equipped with a spiral coil heat exchanger. The results showed that 19.6% of the solidification time was saved by using nanoparticles compared to the case of pure PCM. In addition, nanoparticle dispersion reduced the temperature non-uniformity in the tank. This indicates that the convective heat transfer has decreased because it is well known that convection depends mainly on the movement of the fluid, and the intensity of this movement may diminish with a high dynamic viscosity. It can be concluded that the amount of nanocomposite increases the viscosity, and therefore the PCM fluid cannot move freely.

Hosseinzadeh et al. [19] numerically compared two heat transfer enhancement techniques: adding hybrid-nanoparticles and adding optimized fins into a pure PCM during the discharging process. The comparison proved that the solidification time (discharging time) was less after adding optimized fins compared to dispersing nanoparticles into the PCM. The fins increased the surface of heat exchange and reduced the convective flow of the movement of the liquid PCM, causing enhancement of the performance of the solidification process. Actually, the convective flow is an undesirable factor during the solidification process [8].

Some researchers have studied the coupled effects of using nanoparticles to enhance heat transfer and applying a magnetic field to control the flow. Sheikholeslami and Mahian [20] numerically investigated the influence of magnetic force during the solidification process of an NEPCM. The results showed that, by increasing the Hartmann number from 0 to 10, the solidification rate was improved by 23.5% on average. On the other hand, by adding 4% nanoparticles to the PCM, the solidification rate was augmented by 14%. The results indicated that the magnetic force reduces the velocity of the liquid movement, which is caused by the free convection phenomena, to accelerate the solidification during the heat discharging process. Sheikholeslami [21] conducted a numerical study on the influence of magnetic force on the solidification/discharging process of CuO nanoparticles dispersed in water inside a cylindrical tank with an inner tube. The investigation proved that the solidification rate has a direct relation to the magnetic field and the volume fraction of the nanofluid. The magnetic force can act as a beneficial factor by reducing the molten motion and making the solidification process faster. Sheikholeslami [22] numerically investigated the effects of nanoparticle diameter and volume fraction on the PCM solidification process. The study proved that the solidification rate is enhanced by adding nanoparticles, and the average temperature increases by increasing the concentration and the diameter of the nanoparticles in the PCM. Sheikholeslami [23] performed a numerical study on the behavior of an NEPCM during the discharging process (solidification) inside a cylindrical tank. The work presented the impacts of the inner tube and the volume fractions of CuO nanoparticles. It has been noticed that the shape of the inner tube has a considerable effect on the rate of solidification; in addition, the peak rate of solidification was obtained with dp = 40 nm (nanoparticle diameter). Therefore, the shape of the heat source has an important effect that must be investigated, and it is recommended that the position/shape of the heat source (HTF tube) is investigated, as well as the effect of the shape/dimension/concentration of the dispersed nanoparticles.

Several researchers, such as Milad Sadeghzadeh et al. [24,25], have proposed techniques to control the dynamic viscosity of nanofluids by using an innovative method based on collecting input data such as the nanofluid concentration, the flow rate, and the inlet temperature. They found that the best prediction of thermal performance was obtained by an MLP (Multilayer Perception Artificial Neural Network).

From the above-described literature, it should be mentioned that, in the solidification and melting of Nano-Enhanced Phase Change Materials (NEPCMs), the addition of nanoparticles has a positive effect on the performances of these processes. In addition, several factors are thought to optimize these processes, such as the enclosure geometry; the thermal condition (the position, intensity, etc.); and the nanoparticles diameter, shape, and thermophysical properties.

**Figure 1:**
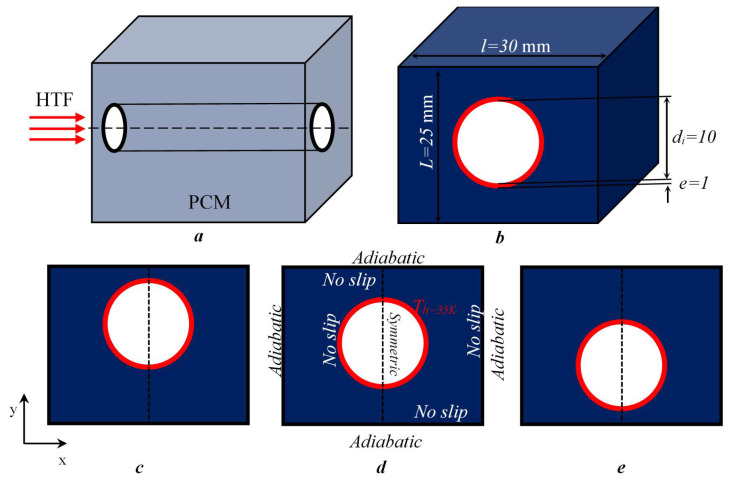
Studied configurations with boundary conditions; (**a**) 3D profile face, (**b**) 3D front face, (**c**) tube at the top position, (**d**) tube at the central position, and (**e**) tube at the bottom position.

The current study was carried out to investigate a new design of NEPCM-filled enclosure (see Figure 1). The influence of the inner-tube position and the benefit of adding Al_2_O_3_ nanoparticles as dispersion to the PCM were investigated. The inner tube had three different vertical positions and the nanoparticle volume fraction was ranged as 0≤ϕ≤0.06. The results are presented and discussed in terms of the liquid–solid fraction, thermal field, flow structure, and Nusselt number. The novelty of this work is based on coupling the influence of varying the position of the heat source (HTF tube) with the addition of the nanoparticles to the PCM capsule.

## 2. Studied Configuration

The studied configurations and the fixed boundary conditions are presented in Figure 1. A cylindrical tube is located inside a rectangular cavity (30/25 mm) filled with NEPCM, and the HTF passes through the tube to ensure the melting of the NEPCM. The present investigation concerns the influence of adding Al_2_O_3_ nanoparticles at various concentrations on the melting process. Three positions of the HTF tube are considered, as presented in Figure 1c,d. The configuration is considered as 2D-axisymmetric to reduce the calculation time. The proprieties of the PCM (n-Octadecane), the nanoparticles (Al_2_O_3_), and the inner-tube wall are presented in Table 1.

## 3. Mathematical Model

The heat transfer during the phase change phenomenon is investigated in this study. The mathematical formulation developed to solve this kind of problem is based on Voller’s model [26]. The enthalpy method based on the Darcy law is combined with the mentioned model by considering the entire domain as a porous medium. The porosity is ranged as: 0≤ε≤1, where, in the liquidus zone, ε=1, in the solidus zone, ε=0, and in the intermediate “mushy” zone, the porosity (liquid fraction) is between 0<ε<1.

By assuming the liquid NEPCM as an incompressible and Newtonian fluid, the governing equations of the present model, including the continuity, modified momentum, and energy in 2D coordinates, are as follow:

Continuity equation

(1) $$\nabla \cdot (\vec{V})=0$$

where $\vec{V}$ is the velocity vector.

Momentum equation

(2) $$\frac{\partial (\rho \vec{V})}{\partial t}+\nabla \cdot (\rho \vec{V})=-\nabla P+\mu \nabla^{2}\vec{V}-S_{a}+S_{b}$$

where ρ is density, μ is dynamic viscosity, and *P* is pressure. $S_{a}$ is the source term of the porosity function [27]. Based on the Carman—Kozeny model [28] and Darcy’s law, $S_{a}$ and ∇(P) are expressed as:

(3) $$S_{a}=-A\;\vec{V}\;\mathrm{and}\;\nabla (P)=-\frac{{\left(1-\eta \right)}^{2}}{\eta^{3}}C\cdot \vec{V}$$

with $A=\frac{{\left(1-\eta \right)}^{2}}{\left(\eta^{3}+10^{-3}\right)}C$.

C is a mushy zone morphology constant and is fixed at 10^6^ kg/m^3^·s [29].

η is the liquid fraction expression of the liquid/solid zone and is used to determine the computed zone, where, in the liquid zone, η=1, in the solid zone, η=0, and in the mushy zone, 0<η<1.

Where

(4) $$\eta (T)=\left\{\begin{array}{l} 0\;\;\;\;if\;T<T_{s}\;\;\;in\;solid\;zone\;\\ 1\;\;\;\;\;if\;T>T_{l}\;\;\;in\;liquid\;zone\\ \frac{T-T_{s}}{T_{l}-T_{s}}\;\;\;if\;T_{l}>T\geq T_{s}\;in\;mushy\;zone \end{array}\right\}$$

where $T_{l}$ and $T_{s}$ are the liquid and solid temperature of the PCM, respectively.

By using: ($T_{s}=T_{m}-\Delta T$) and ($T_{l}=T_{m}+\Delta T$), where $T_{m}$ is the melting point of the PCM and ΔT is the melting temperature range (ΔT=2K), the liquid fraction expression becomes:

(5) $$\eta (T)=\left\{\begin{array}{l} 0\;\;\;\;\;\;\;if\;T<(T_{m}-\Delta T)\\ 1\;\;\;\;\;\;\;\;\;if\;T>(T_{m}+\Delta T)\\ \frac{\left(T-T_{m}+\Delta T\right)}{2\Delta T}\;\;\;if\;(T_{m}+\Delta T)>T\geq (T_{m}-\Delta T) \end{array}\right\}$$

where Equation (5) is used as a conditional equation in Comsol-Multiphysics.

Based on the Darcy law, the momentum equation for a fluid crossing a porous medium is expressed as:

(6) $$\vec{V}=\frac{\kappa }{\mu }\nabla P$$

where κ is the permeability, which depends on the liquid fraction, η, and $S_{b}$ is the Boussinesq approximation of thebuoyancy force:

(7) $$S_{b}=\rho_{l}\beta (T-T_{m})\vec{g}$$

where $\rho_{l}$ is the density of the liquid NEPCM, β is the thermal expansion coefficient, and $\vec{g}$ is the gravity acceleration vector.

Energy equation

(8) $$\frac{\partial (\rho H)}{\partial t}+\nabla \cdot (\rho \vec{V}H)=\nabla \cdot (k\nabla T)$$

where *H* is the specific enthalpy:

(9) H=h+ΔH

and h is the sensible enthalpy defined as:

(10) $$h=h_{ref}+\int_{T_{ref}}^{T}c_{p}dT$$

where k, $h_{ref}$, $T_{ref}$, $c_{p}$, and ΔH are, the thermal conductivity, reference sensible enthalpy, reference temperature, specific heat capacity, and melting latent heat, respectively.

The effective properties of the NEPCM dispersion are evaluated as follow [15]:

Thermal conductivities of the Liquid NEPCM ($k_{nl}$) and the Solid NEPCM ($k_{ns}$):

(11) $$k_{nl}=\frac{k_{np}+2k_{l}-2(k_{l}-k_{np})\phi }{k_{np}+2k_{l}-(k_{l}-k_{np})\phi }k_{l}+5.10^{4}\beta_{0}\phi {\left(\rho c_{p}\right)}_{l}\sqrt{\frac{K_{b}T}{\rho_{np}d_{p}}}f(T,\phi )$$

(12) $$k_{ns}=\frac{k_{np}+2k_{s}-2(k_{s}-k_{np})\phi }{k_{np}+2k_{s}-(k_{s}-k_{np})\phi }k_{s}+5.10^{4}\beta_{0}\phi {\left(\rho c_{p}\right)}_{l}\sqrt{\frac{K_{b}T}{\rho_{np}d_{p}}}f(T,\phi )$$

where $k_{np}$, $k_{l}$ and $k_{s}$ are, respectively, the thermal conductivity of the nanomaterial, the liquid PCM, and the solid PCM, and $\rho_{np}$ is the density of the nanomaterial.

With:

(13) $$f(T,\phi )=(2.817\cdot 10^{-2}\phi +3.917\cdot 10^{-3})\frac{T}{T_{0}}+(-3.0669\cdot 10^{-2}\phi -3.91123\cdot 10^{-3})$$

By considering that the properties of the NEPCM within the liquid/solid zone are constant and temperature-independent, the function (*f*) is evaluated at *T* = 320 K and *T*_0_ = 273 K [15], where: $\beta_{0}=8.4407{\left(100\phi \right)}^{-1.07304}$, $K_{b}=1.381\times 10^{-23}$ is the Boltzmann constant, and *d_p_* is the nanoparticles diameter.

Densities of the Liquid ENPCM ($\rho_{nl}$) and the Solid ENPCM ($\rho_{ns}$):

(14) $$\rho_{nl/s}=(1-\phi )\rho_{s/l}+\phi \rho_{np}$$

where $\rho_{l}$ and $\rho_{s}$ are, respectively, the density of the nanomaterial: the liquid PCM and the solid PCM.

Specific heats of the Liquid ENPCM $c_{pnl}$ and the Solid ENPCM ($c_{pns}$):

(15) $${\left(\rho \cdot C_{p}\right)}_{nl/s}=\phi \cdot {\left(\rho \cdot C_{p}\right)}_{np}+\left(1-\phi \right)\cdot {\left(\rho \cdot C_{p}\right)}_{nl/s}$$

where $c_{pnp}$, $c_{pl}$, and $c_{ps}$ are, respectively, the specific heat of the nanomaterial, the liquid PCM, and the solid PCM.

Dynamic Viscosity of the Liquid ENPCM ($\mu_{nl}$):

(16) $$\mu_{nl}=0.983e^{12.959\phi }\mu_{l}$$

where $\mu_{l}$ is the dynamic viscosity of the liquid PCM.

Latent heat of fusion of the ENPCM ($\Delta H_{nl}$):

(17) $$\Delta H_{nl}=(1-\phi )\Delta H\rho_{l}/\rho_{nl}$$

where ΔH is the latent heat of fusion of the pure PCM.

Thermal expansion coefficient of the Liquid ENPCM ($\beta_{nl}$):

(18) $${\left(\rho \cdot \beta \right)}_{nl}=\phi \cdot {\left(\rho \cdot \beta \right)}_{np}+\left(1-\phi \right)\cdot {\left(\rho \cdot \beta \right)}_{l}$$

where $\beta_{np}$ is the thermal expansion coefficient of nanoparticle.

The density variation of the NEPCM:

(19) $$\rho (T)=\rho_{ns}+(\rho_{nl}-\rho_{ns})\cdot \eta (T)$$

The specific heat of the NEPCM:

(20) $$c_{p}(T)=c_{pns}+(c_{pnl}-c_{pns})\cdot \eta (T)+D\cdot \Delta H(T)$$

where *D* is the smoothed Delta Dirac function: $D=e^{\left(-{\left(T-T_{m}\right)}^{2}/\Delta T^{2}\right)}/\left(\Delta T\sqrt{\pi }\right)$ [8,29].

The thermal conductivity of the NEPCM:

(21) $$k(T)=k_{ns}+(k_{nl}-k_{ns})\cdot \eta (T)$$

### 3.1. Mesh Independency Study

Table 2 presents the mesh dependency test. The average liquid fraction, average temperature, and average velocity at t = 3000 s are chosen as the sensitive variables.

For all the considered geometrical configurations (inner tube at the top, central, and bottom positions) the chosen meshes are: 26,770, 27,107, and 26,794 elements, respectively. These amounts of elements were chosen to ensure results accuracy and computational time economy.

**Figure 2:**
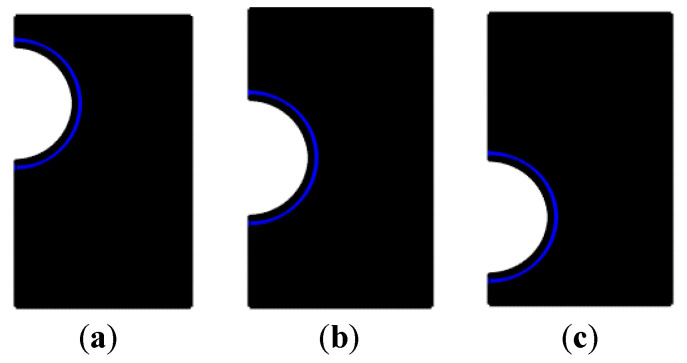
The computation grids ((**a**) top position, (**b**) central position, and (**c**) bottom position).

The computation grids for the considered cases are displayed in Figure 2. It is observed that the grid of the simulation area is well-refined in order to define the mushy zone transition at each time step [30].

### 3.2. Solution Procedure

The fixed-grid approach has proved its superiority in studying natural convection effects during phase change problems. Actually, it does not require any explicit treatment of the moving boundary; furthermore, it gives a more realistic and consistent solution to the studied problems [31,32,33].

The resolution procedure is performed using the constant Newtonian iteration technique (Six iterations for each time step, and the damping factor is set at 0.9; the time step is selected automatically to reach the defined error tolerance, and “0.001” is used as a tolerance value). The Backward Euler method is used as a time marching method (the backward order is between 1 and 2).

### 3.3. Model Validation

**Figure 3:**
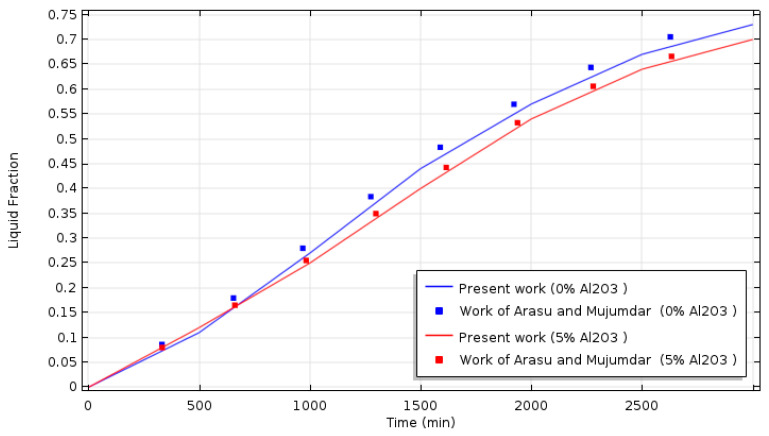
Temporal variation of the liquid fraction: comparison with numerical results of Arasu and Mujumdar [12].

A validation test was managed by comparing the present model and previously published results. Figure 3 presents the comparison of the liquid fraction obtained by the present model and the liquid fraction of the work of Arasu and Mujumdar [12]; the comparison is showing an acceptable agreement with the findings of reference [12].

## 4. Results and Discussion

### 4.1. Liquid Fraction, Thermal Field, and the Streamlines at Deferent Time Step

**Figure 4:**
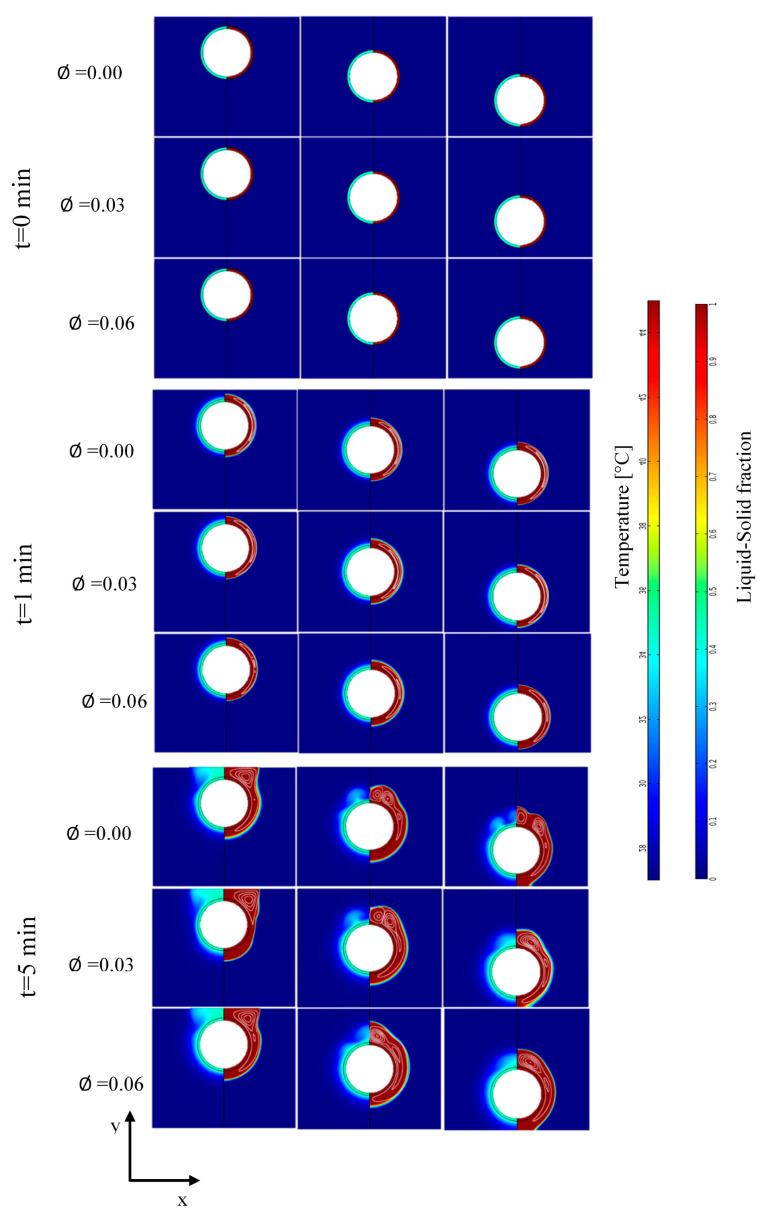

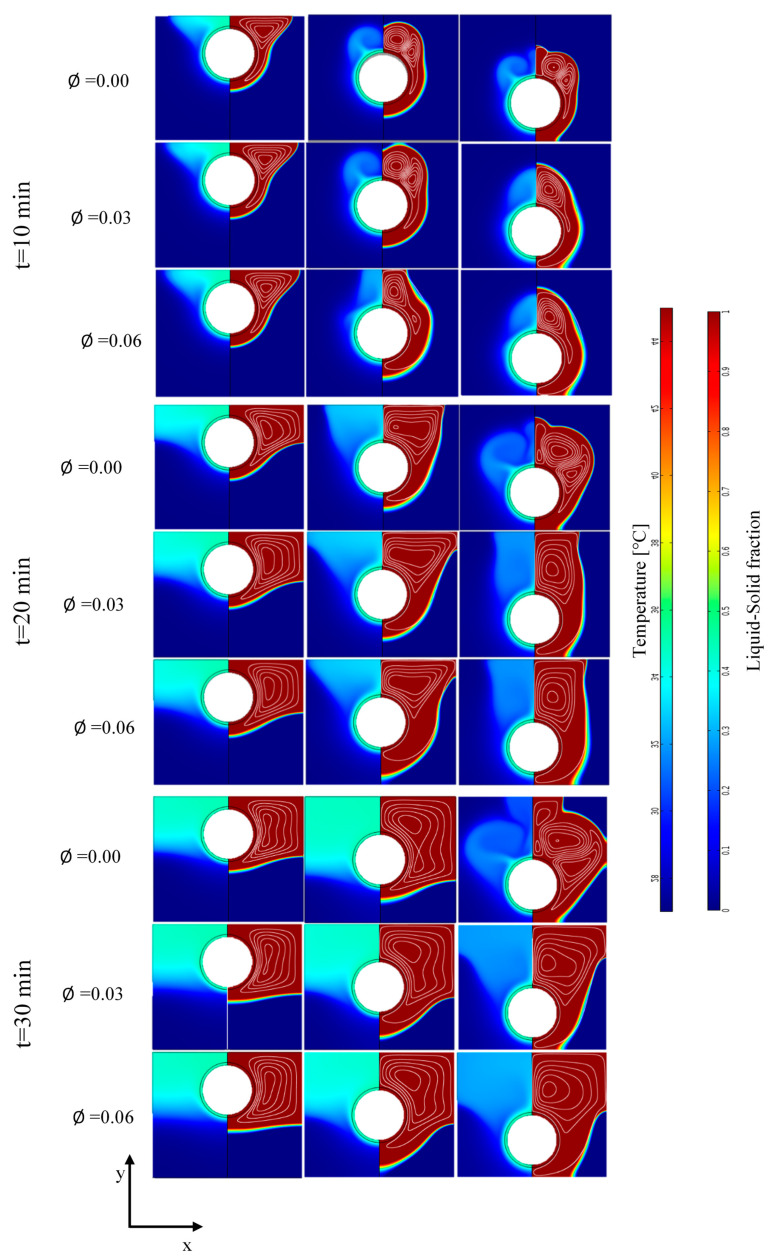

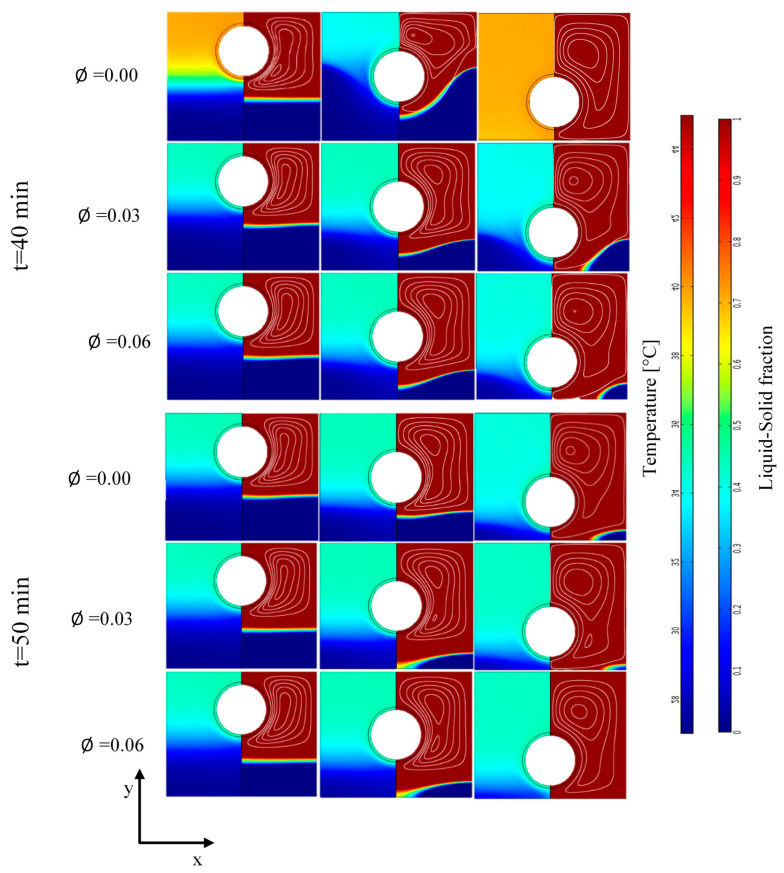
Contour plots of the liquid zone, the streamlines (right section), and the thermal field (left section).

Plots of the liquid fraction, thermal field, and streamlines at different time steps (t = 0, 1, 10, 20, 30, 40, and 50 min) with different nanoparticles concentrations (ϕ = 0.00, 0.03, and 0.06) and for the three considered geometrical configurations (top, center, and bottom positions of the tube) are shown in Figure 4. The results show that the liquid fraction of all the nanoparticle volume fractions and the different positions of the inner tube behave generally in the same way. The liquid fraction begins to grow from the inner tube towards the adiabatic walls.

In addition, the results in Figure 4 show that the thermal distribution follows a behavior similar to that of the liquid fraction, i.e., the temperature increases from the inner tube to other parts of the container. The thermal field corresponds well to the growth of the liquid–solid fraction and to the position of the mushy zone. The thermal field and the liquid fraction take a regular shape (parallel to the shape of the inner tube) at the early stages of the melting process and change to an irregular shape at the later stages of the process. The thermal field and the mushy zone show up in a wavy form in advanced stages of the process due to the convective effects. Actually, the heat transfer is dominated by the conduction mode in earlier phases.

Streamlines in Figure 4 show the flow structure inside the liquid PCM. It is noticed that the molten PCM moves in rotating cells due to the thermal gradation effect that causes the hot parts of the liquid PCM to rise and the colder parts to fall due to the buoyancy force.

The progress of the melting process is enhanced compared to the pure PCM case (ϕ = 0.00) by the addition of nanoparticles. Indeed, the melting process accelerates with the increase of the nanoparticle’s concentration for all the considered positions of the inner tube.

At t = 1 min, the shapes of the thermal field and the liquid zone are formed regularly according to the shape of the inner tube, for all the geometrical cases and all the nanoparticles volume fractions. In addition, the streamlines follow trajectories that are parallel to the inner tube. When the time is up, the streamlines take a circular shape, and the flow intensity of the molten PCM becomes more significant; thus, the shape of the molten zone in the advanced stage has a wavy shape.

At 40 min and 50 min, for the top-position tube, the melt reaches an area under the inner tube characterized by a regular shape due to the dominance of the conductive heat transfer heat mode. By increasing the nanoparticle concentration, the heat transfer is enhanced, especially when it is controlled by the conduction mode. In other words, during the initial time of the melting process, the role of the nanoparticles is more pronounced, allowing a better heat distribution; consequently, the melting process becomes faster.

### 4.2. Average Nusselt Number

**Figure 5:**
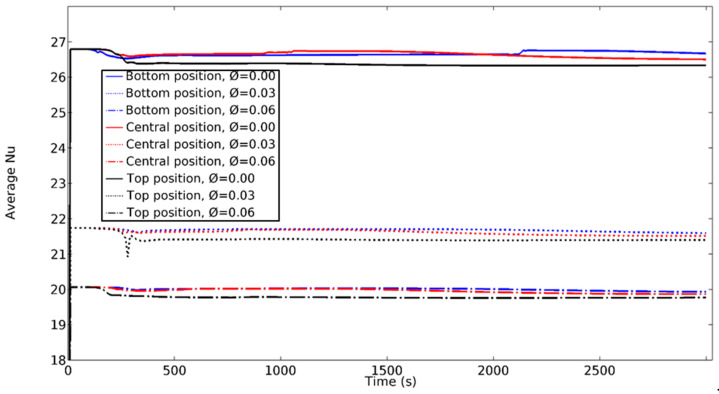
Temporal variation of the average Nusselt for the considered configurations and different nanoparticle concentrations.

Figure 5 shows the temporal evolution of the average Nusselt number ($\bar{Nu}$) on the outer wall of the tube for all the considered positions of the tube and the different concentrations of nanoparticles (ϕ = 0, 0.03, and 0.06).

The evolution of Nusselt takes the same behavior for all the considered configurations, where the Nusselt $\bar{Nu}$ increases sharply after few seconds from the running time to reach a peak value, and then becomes quasi-constant until the end of the process. $\bar{Nu}$ reaches the highest values with the cases of nanoparticles concentration equal to 0.00 (pure PCM) with all tube position cases and it takes the lowest values with the case of Nano concentration is ϕ = 0.06. In addition, the Nusselt values are increased by moving the inner tube downwards. As well known the Nusselt number reflects the intensity of the convection compression to conduction, from this figure it is clear that the convective heat transfer dominates the conduction since the early stage of the melting process, besides the convection effect increases as the inner tube is moved to the bottom section of the tank.

### 4.3. Liquid Fraction Development Curves of All Cases

**Figure 6:**
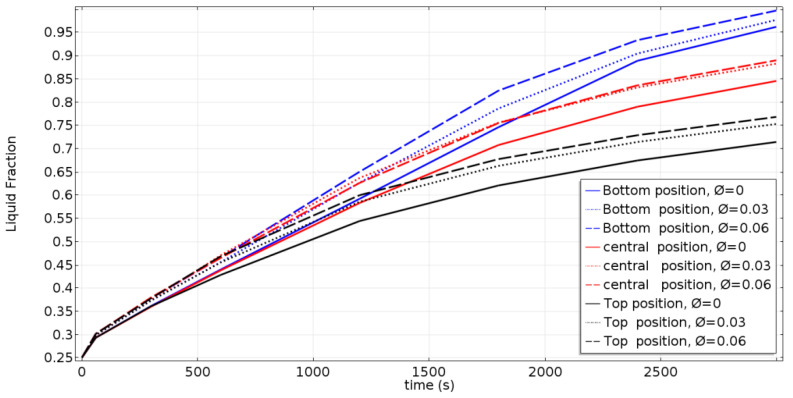
Liquid fraction development.

Figure 6 depicts the temporal variation of the molten fraction, including the effects of the nanoparticle concentration and the inner-tube position. It is clear that the molten fraction increases with time in all cases. The blue, red, and black curves refer to the location of the inner tube, i.e., the bottom, central, and top positions, respectively. The melting time decreases as the inner tube is moved to the bottom of the cavity, and the melting time decreases by increasing the nanoparticle concentration due to the enhancement of the heat transfer. At the beginning (t < 500 s) of the melting process, the effect of adding nanoparticles is not very effective on the melt fraction. When the time is up, the liquid fraction is considerably increased, especially for higher nanoparticle concentrations. This is an interesting result, because the addition of low-cost nanoparticles, such as Al_2_O_3_, will lead to the amelioration of the charging process performances. In fact, for all the considered inner-tube positions, the melt fraction is approximatively 10% higher for ϕ = 0.06 (NEPCM) compared to the pure PCM.

### 4.4. Stored Thermal Energy

**Figure 7:**
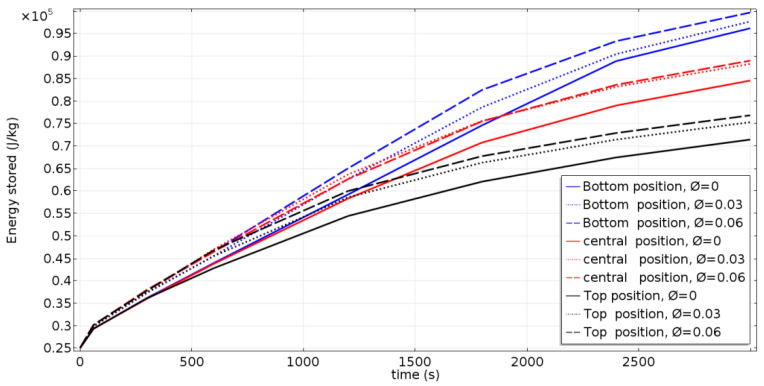
Temporal evolution of the stored energy.

Figure 7 shows the temporal variation of the thermal energy stored by the NEPCM. The stored energy increases with time in all cases, as presented in Figure 7. It can also be noted that the energy storage rate follows the same pattern of evolution of the melting rate. The rate of charge of stored energy increases with the displacement of the inner tube towards the bottom of the cavity, and with the increase of the concentration of nanoparticles. This improvement is due to the increased heat distribution in the NEPCM.

## 5. Concluding Remarks

This numerical study aimed to investigate the effect of adding Al_2_O_3_ nanoparticles to a PCM-filled cavity equipped with an inner HTF tube. The influence of the position of the internal tube on thermal behavior has also been studied. The main findings of the study can be summarized as follows:

- The melting rate increases as the position of the inner tube changes towards the bottom section of the cavity; this is because the upper region is larger and free convection is the dominating heat transfer mode in this region. In addition, conduction is the dominating mode in the lower region, especially at the beginning of the melting process. Therefore, the amount of stored energy increases as the influence of convection increases with the displacement of the inner tube downwards.
- The melting and charging rates are improved by increasing the volume fraction of the nanoparticles and, due to the enhancement, the thermal conductivity that ameliorates the heat transfer rate.
- The temporal evolutions of the melting process and the amount of stored energy are quasi-similar at the start of the process because the heat input was managed by conduction. However, at later stages of the process, the heat transfer becomes dominated by convection.

## Figures and Tables

**Table 1:** Thermophysical proprieties of n-Octadecane (PCM),inner-tube wall, and Al_2_O_3_ nanoparticles [15].

Properties of the Materials	PCM		Inner-Tube Wall	Nanoparticle
	(n-Octadecane)		Copper	Al_2_O_3_
	Solid	Liquid		(*d_p_* = 59 × 10^−9^ m)
thermal conductivity, k, (W/m K)	0.39	0.157	401	36
density, ρ, (kg/m^3^)	814	770	8900	3600
dynamic viscosity, μ, (Pa s)	-	3.8 × 10^−3^		
thermal expansion coefficient, β, (1/K)	-	8.5 × 10^−4^		7.8 × 10^−6^
specific heat, $c_{p}$, (kJ/kg K)	1.9	2.2	0.385	0.765
the melting point of the PCM, $T_{m},$ (K) (28.05 °C) and its latent heat of fusion, ΔH, (241 kJ/kg)				

**Table 2:** Grid sensitivity tests.

Total Number of Elements		Average Liquid Fraction (t = 3000 s)	Average Temperature (at t = 3000 s) [K]	Average Velocity (at t = 3000 s) [m/s]
Top position	11,949	0.61601	302.97	2.0014 × 10^−5^
Central position	11,976	0.73455	304.45	2.4125 × 10^−5^
Bottom position	12,014	0.81541	305.58	0.6457 × 10^−4^
Top position	26,770	0.71434	304.03	2.5427 × 10^−5^
Central position	27,107	0.84562	305.52	3.8615 × 10^−5^
Bottom position	26,794	0.96204	306.51	1.5423 × 10^−4^
Top position	69,191	0.71454	304.05	2.54235 × 10^−5^
Central position	70,172	0.84581	305.57	3.8623 × 10^−5^
Bottom position	69,151	0. 96245	306.54	1.5434 × 10^−4^

## Nomenclatures

**Symbols**	
$c_{p}$	Specific heat, (J/kg K)
g	Gravitational acceleration, (m s^−2^)
k	Thermal conductivity, (W/m K)
P	Pressure, (Pa)
T	Temperature, (K)
$T_{m}$	Melting Point, (K)
$T_{l}$	Liquid temperature, (K)
$T_{s}$	Solid temperature, (K)
$T_{in}$	Initial temperature, (K)
ΔT	Transition temperature range, (K)
t	Time, (s)
$\vec{V}$	Velocity Vector, (m s^−1^)
u	Velocity in x-direction, (m s ^−1^)
v	Velocity in y-direction, (m s^−1^)
h	Sensible enthalpy, (kJ/kg)
H	Total enthalpy, (kJ/kg)
ΔH	Latent heat, (kJ/kg)
$h_{ref}$	Enthalpy reference, (kJ/kg)
D	Smoothed Delta Dirac function
$S_{a}$	Source term of porosity function, (kg.s^2^.m^−2^)
$S_{b}$	Boussinesq approximation of buoyancy force, (kg.s^2^.m^−2^)
C	Mushy region morphology constant, (kg/ m^3^.s)
dp	Nanoparticle diameter, (m)
Kb	Boltzmann constant
**Greek letters**	
β	Thermal expansion coefficient, (K^−1^)
μ	Dynamic viscosity, (Pa.s)
κ	Permeability, (m^2^)
ε	Porosity (liquid fraction)
η	Porosity function
ρ	Density (kg/m^3^)
ϕ	Nanoparticle concentration
**Subscript**	
s	Solid state
l	Liquid state
n	Nanoparticle
**Abbreviations**	
PCM	Phase Change Material
HTF	Heat Transfer Fluid
NEPCM	Nanoparticle Enhanced Phase Change Material
TES	Thermal Energy Storage
